# *Ectoplana limuli*, a Parasite of the Horseshoe Crab (*Tachypleus tridentatus*): A New Record in China

**DOI:** 10.3390/biology14101412

**Published:** 2025-10-14

**Authors:** Zimeng Luo, Lingtong Ye, Ziwei Ying, Chenxiang Deng, Xiaoyong Xie, Xiaohai Chen, Ting Li

**Affiliations:** 1 Key Laboratory of South China Sea Fishery Resources Exploitation & Utilization, Ministry of Agriculture and Rural Affairs, South China Sea Fisheries Research Institute, Chinese Academy of Fisheries Sciences, Guangzhou 510300, China; luozimeng522@163.com (Z.L.); lingtong2753@126.com (L.Y.); dcx29743517@163.com (C.D.); seanjoshua@163.com (X.C.); liting@scsfri.ac.cn (T.L.); 2 Guangdong Provincial Key Laboratory of Fishery Ecology and Environment, South China Sea Fisheries Research Institute, Chinese Academy of Fisheries Sciences, Guangzhou 510300, China; 3 College of Fisheries and Life Science, Shanghai Ocean University, Shanghai 201306, China; 4 College of Sorbonne University, 75005 Paris, France; 5 Sanya Tropical Fisheries Research Institute, Sanya 572019, China

**Keywords:** Limulidae, 18S rDNA, Tricladida

## Abstract

The mortality rate of first- to second-instar horseshoe crabs during molting is extremely high under culture conditions (pH of 7.6 ± 0.1, salinity of 27 ± 2, temperature of 26–32 °C), and we preliminarily speculate that it is related to disease. Our team found that *Ectoplana limuli* was attached to the ventral limbs of adult horseshoe crabs during culture. Parasite samples were collected from the external appendages and mouthparts of adult *Tachypleus tridentatus* for classification and identification. The primary objective of this experiment was to identify the species of this parasite and determine its taxonomic status. To this end, the experiment employed a combination of morphological methods and 18S rDNA gene molecular markers. The obtained sequences showed over 99% homology with *Ectoplana limuli*. Sequence alignment and phylogenetic tree results indicated that *Ectoplana limuli* showed a closer genetic relationship with *Nerpa fistulata*, but more distant relationships with *Paucumara* and *Baikalobia*. This is the first time that this parasite has been found in China, providing additional information for the study of horseshoe crab diseases.

## Simple Summary

Disease can contribute to the mortality of the horseshoe crab during molting. We discovered an ectoparasite on the body surface and mouthparts of adult *Tachypleus tridentatus*. To identify the species and its taxonomic status, we conducted a study that combined morphological methods with 18S rDNA molecular markers. The results showed that the parasite is highly homologous to *Ectoplana limuli*. This study firstly reports this parasite in China, providing important insights for disease control and contributing to the conservation of *T. tridentatus* aquaculture.

## 1. Introduction

*Ectoplana limuli* (Ijima & Kaburaki, 1916) is a marine species of the family Uteriporidae (Platyhelminthes: Tricladida), first found on *Tachypleus tridentatus* (Leach, 1819) [1]. It was initially discovered in the inland sea region of Hyogo Prefecture, Japan, and has been recorded in the Gulf of Thailand as well as in other localities in Japan [2]. *Ectoplana limuli* is an ectosymbiotic organism, primarily parasitizing the gills of horseshoe crabs (such as *Tachypleus tridentatus*, *Tachypleus gigas*, and *Carcinoscorpius rotundicauda*) [1]. Research on the diversity of Tricladida in China is relatively limited [3]. According to studies by Japanese experts, *Dugesia japonica* is currently the only known species from China [4]. Both *Dugesia* and *Ectoplana* belong to the order Tricladida (Platyhelminthes), making them taxonomically related groups, which provides a comparative reference for our study on *Ectoplana limuli*. There are no research reports on *Ectoplana limuli* in China. Accurate identification of parasitic species is crucial for epidemiology, prevention, and treatment [5]. Molecular biology techniques, such as PCR, have been widely applied in the field of parasitology, particularly in molecular taxonomy and species identification of parasites, with significant progress [6]. The knowledge of parasites of *T. tridentatus* from China is scarce, and it is hard to achieve accurate diagnosis and effective prevention and treatment.

*Tachypleus tridentatus* is distributed in the intertidal zone of Guangdong, Guangxi, and Hainan in China, as well as in the southern waters of Japan and Southeast Asia. Its ecological significance and economic value are highly regarded [7,8,9]. The blood of *T. tridentatus* can be used to produce Limulus amebocyte lysate (LAL), an essential quality control reagent in biomedical products.

Fossil records of horseshoe crabs date back to the early Paleozoic Ordovician period, approximately 475 million years ago [10]. In 2012, the IUCN explicitly stated that horseshoe crabs play a critical role in maintaining the normal functioning of ecosystems and, as a natural resource, require sustained and effective management [11]. At present, the abundance of horseshoe crabs has dropped sharply in China. The protection and breeding of this species have become urgent issues that need to be addressed [12,13]. At the same time, when breeding juvenile horseshoe crabs, our team found extremely high mortality during the period from molting at the 1st instar to the 2nd instar. We initially speculated that it was related to disease. Infectious diseases affecting horseshoe crabs include those caused by algae, fungi, bacterial colonies, filamentous cyanobacteria, Gram-negative bacteria, and various parasites [14,15]. Current research on aquatic animal diseases has yielded extensive reports on their etiology and prevention techniques concerning economically important fish species [16], yet studies on diseases affecting protected animals such as *T. tridentatus* remain scarce. This research imbalance directly hinders the development of conservation strategies for endangered species. This abnormally high mortality in *T. tridentatus* breeding still lacks a scientific explanation, with speculation linking it to disease and nutrition. Studies have identified multiple parasites in horseshoe crabs, including trematodes, nematodes, and flatworms [17]. Horseshoe crabs are frequently affected by ciliates (such as *Pananophrys* spp.), flagellates (such as *Hexamita* spp.), and amoebas (family *Paramoebidae*) [17]. In addition to factors such as overharvesting and environmental pollution, certain diseases may also contribute to their population decline. Therefore, there is an urgent need to identify the species of parasites found on the external surfaces of *T. tridentatus* and to develop effective prevention and control measures to block their transmission pathways.

In this study, field sampling and prevalence statistics were conducted on *T. tridentatus* individuals from two sources: those in aquaculture ponds where parasitic infections were found, and those accidentally captured during field surveys from natural habitats (such as coastal intertidal zones) that also had parasitic infections. The collected parasites were identified through histology, electron microscopy, and molecular analysis, confirming the parasite species as *Ectoplana limuli*. This study is the first discovery of a Tricladid parasitizing the horseshoe crab in China, providing additional literature references for subsequent research on horseshoe crab diseases, including the development of prevention and control measures. Conducting such fundamental research will not only fill knowledge gaps in China’s understanding of Tricladida parasites but also provide new insights into the mechanisms of *T. tridentatus* population decline.

## 2. Materials and Methods

### 2.1. Sample Collection and Management

The parasite samples were collected from the external surfaces of the ventral limbs of 15 adult cultured and wild specimens of *T. tridentatus*. Parasite samples were collected from 12 crabs (three infected individuals per aquaculture pond and four ponds in total) at the Zhanjiang Breeding Base of the South China Sea Fisheries Research Institute, Chinese Academy of Fishery Sciences. The size of the aquaculture pond is 4 m × 4 m × 0.8 m. The culture conditions of horseshoe crabs are as follows: pH of 7.7 ± 0.1, salinity of 28 ± 2, temperature of 30 ± 2 °C, and dissolved oxygen concentration of 7.01 ± 0.96 mg/L, with a natural photoperiod (12 L:12 D). Additionally, parasites were sampled from three specimens of *T. tridentatus* captured during the field resource survey conducted in the Liuheng Sea area of Zhoushan, Zhejiang Province. We collected parasites and then released these horseshoe crabs back into the sea. All samples were collected using sterilized tweezers and were placed in sterile centrifuge tubes containing anhydrous ethanol and paraformaldehyde for fixation, preparing them for subsequent experimental analysis.

### 2.2. Prevalence Analysis

Referring to [18], prevalence is defined as the number of hosts infected with parasites in a host sample relative to the total number of hosts in that sample.

### 2.3. Parasite Morphological Observation

Observations were conducted under a Leica DM4B/DFC7000T upright microscope, and measurements and imaging were performed using a computer-based image analysis system and NIS Elements D software (v2.20). The parasite body length and weight were measured using the integrated digital measurement tool of a Leica DFC320 Digital Microscope Camera (Leica Microsystems, Wetzlar, Germany) on a total of 15 specimens (12 from farmed and 3 from wild adult *T. tridentatus*). Data from all specimens were pooled for analysis to obtain the mean and standard deviation. The morphological features of the anterior and posterior ends of the parasites were examined, and key parameters (including external appearance, shape, length, and coloration) were measured for subsequent analysis.

### 2.4. Histological Observation

Tissue sections were prepared according to the standard operating procedures (SOPs) for pathological tissue sampling, fixation, embedding, paraffin sectioning, and frozen sectioning, as outlined by Wuhan Servicebio Technology Co., Ltd., Wuhan, China [19].

Next, the sections were immersed in a high-definition constant staining pre-treatment solution for 1 min, then stained in hematoxylin staining solution for 3–5 min, rinsed with tap water, differentiated in differentiation solution, blued in bluing solution, and then rinsed under running water [19]. Subsequently, after dehydration in 95% alcohol for 1 min, the sections were immersed in eosin staining solution for 15 s. Next, the sections were sequentially passed through absolute ethanol I, II, and III (2 min each), n-butanol I and II (2 min each), and xylene I and II (2 min each) for transparency, and then mounted with neutral resin [19]. Finally, under the microscope, the nuclei appeared blue, and the cytoplasm appeared red. The experiment was conducted by Wuhan Servicebio Technology Co., Ltd. [19].

### 2.5. Molecular Biological Identification

Approximately 30–50 mg of parasite tissue fixed in anhydrous ethanol was placed into a new 2 mL centrifuge tube and left in a fume hood to accelerate ethanol evaporation. Subsequently, DNA extraction was performed following the instructions of the Mollusk Tissue DNA Extraction Kit (Meiji Biotech, Guangzhou, China). First, the SSU rDNA was amplified using universal eukaryotic primers MedlinA (CGTGTTGATCCTGCCAG) and MedlinB (TGATCCTTCTGCAGGTTCACCTAC) [20], which were synthesized by Tsingke Biological Technology Co., Ltd, Beijing, China. The PCR amplification system was 25 μL, including 1 μL of forward and reverse primers (10 μmol/L each), 2 μL of template DNA, 12.5 μL of PCR Mix (Dongsheng, Guangzhou, China), and 8.5 μL of sterilized double-distilled water. The amplification program was as follows: pre-denaturation at 94 °C for 5 min; 30 cycles of denaturation at 94 °C for 1 min;, annealing at 55 °C for 1 min, and extension at 72 °C for 1 min; followed by a final extension at 72 °C for 10 min. PCR products were detected by 1% agarose gel electrophoresis, and sequencing was performed by Tsingke Biological Technology Co., Ltd. after the target bands appeared. Since universal eukaryotic primers may not accurately distinguish certain closely related species or specific taxa, specific primers were designed to improve identification accuracy [21]. Primer 6.0 software was used to batch-design forward and reverse primer sequences targeting SSR sites [22], with primer lengths of 18–22 bp, Tm values of 57–63 °C, and annealing temperature differences between forward and reverse primers not exceeding 3 °C, resulting in product lengths of 90–250 bp. The specific primer sequences were F: ATTGTCGGTGTCTTCGTTA and R: TTGTTCGTCTGCTGATGAT, synthesized by Tsingke Biological Technology Co., Ltd. The PCR amplification conditions were the same as above, and the products were detected by 1% agarose gel electrophoresis. Sequencing was performed by Tsingke Biological Technology Co., Ltd. after the target bands appeared.

To ensure sequence accuracy, the sequences were manually corrected based on the sequencing chromatograms. The sequencing results were subjected to BLAST (version 2.14.0) comparison on NCBI, and repetitive sequences were identified using the Gene Repeat Sequence Calculator (https://blast.ncbi.nlm.nih.gov/Blast.cgi, accessed on 24 August 2024). Similarity analysis was performed using Mega (version 11.0). Sequences of the species to be identified were searched in the GenBank database, and the similarity between the target sequences and the sequences of the species to be identified in the database was compared [23].

### 2.6. Phylogenetic Analysis

Sequences were aligned with those reported in GenBank using NCBI BLAST (version 2.14.0) with default parameters, and the alignment was further refined using ClustalW (version 2.1) with gap opening penalty 10 and gap extension penalty 0.1. A total of 56 homologous sequences (GenBank accession Nos.: MH916612.1, MH916611.1, MH916610.1, MH916609.1, MT994584.1, MH916614.1, MZ305322.1, KY848667.1, MZ305317.1, MZ305318.1, MH916615.1, MH916616.1, MZ305311.1, MZ305323.1, MZ305326.1, D85088.1, MH916613.1, MT994585.1, DQ665998.1, AF013149.1, MH108587.1, Z99948.1, OQ034301.1, OR758633.1) with >95% nucleotide similarity were retrieved from GenBank for subsequent analysis. The phylogenetic tree was constructed using the Neighbor-Joining method in MEGA 11 software, based on the Kimura 2-parameter nucleotide substitution model (as determined by ModelTest within MEGA 11). Reliability of the tree topology was evaluated by bootstrap analysis with 1000 replicates [24].

The phylogenetic tree analysis was conducted using the Neighbor-Joining method [25]. The optimal phylogenetic tree the numbers in parentheses represent the percentage of replicate trees in which the associated taxa clustered together in the bootstrap test (1000 replicates) [26]. The tree is drawn to scale, with branch lengths in the same units as those of the evolutionary distances used to infer the phylogenetic tree. The evolutionary distances were computed using the Poisson correction method [27] and are in the units of the number of amino acid substitutions per site. This analysis involved 24 amino acid sequences. All positions containing gaps and missing data were eliminated from the dataset (pairwise deletion option). There were a total of 361 positions in the final dataset. Evolutionary analyses were conducted in MEGA11 [28].

## 3. Results

### 3.1. Prevalence

**Figure 1:**
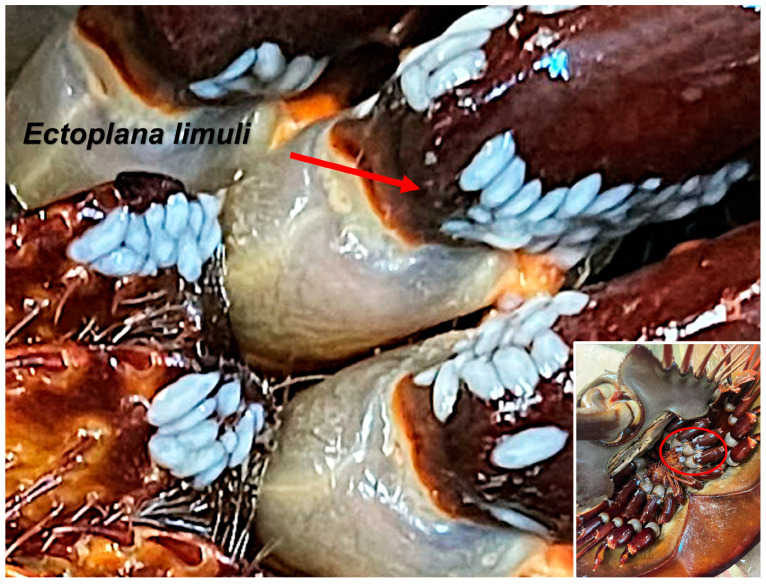
*Tachypleus tridentatus* infected with parasites. The parasite is the white rice-like object circled in a red oval.

Each pond contained 24 individuals. Among the four ponds, the number of *T. tridentatus* found with attached parasites was 12, 16, 8, and 0, respectively. The prevalence of infection was 50%, 67%, and 33%, respectively. The infection sites were primarily around the appendages and mouthparts (Figure 1). The gill lamellae of *T. tridentatus* had decayed.

### 3.2. Morphological Observation

**Figure 2:**
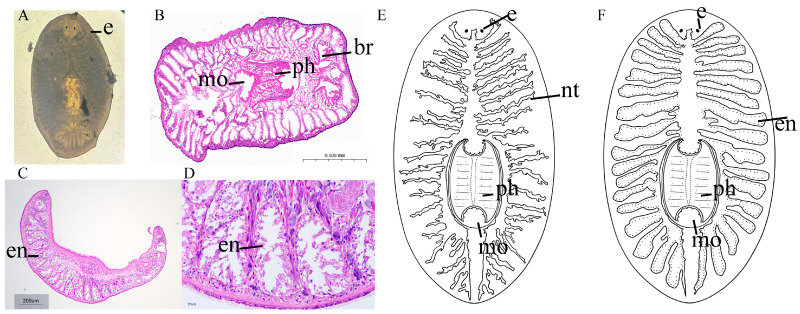
General morphology and internal anatomy of *Ectoplana limuli*-infected gill lamellae of *T. tridentatus*. (**A**) Whole worm, dorsal view; (**B**) Cross-section of the anterior part of the body; (**C**) Sagittal section; (**D**) Enteric canal; (**E**) Diagram of the nervous system; (**F**) Diagram of the digestive system. br: brain; e: eye; en: enteric canal; mo: mouth; ph: pharynx; nt: nerve tissue. The parasite species is *Ectoplana limuli*, the infected host is *Tachypleus tridentatus*, and the discovery site is the gill lamellae of *T. tridentatus*.

The parasite has a semi-circular shape at both the head and tail, with an overall shape that is a long ellipse and flattened form. It possesses a pair of eyes located on the dorsal side of the head, which are deep black in color. The brain is situated between the two eyes (Figure 2A). Hematoxylin-eosin staining section results showed an overall oval cross-section, with a distinct boundary of the cuticular body wall. Below the body wall lies the muscle layer, and beneath the muscle layer is the nerve tissue. The digestive system consists of a mouth, pharynx, and intestinal branches (Figure 2B–D). The mouth is located on the ventral side of the mid-body. The mouth is situated at the posterior end of the pharynx, which is located centrally on the ventral side of the body, and has a robust muscular structure. The intestinal branches are all located on the dorsal side of the body. The body width is 1000 ± 300 μm, and the body length is 1600 ± 300 μm. Figure 2E,F are schematic representations of *Ectoplana limuli*.

### 3.3. Molecular Identification

Fifteen 18S rDNA gene fragment sequences (PX417341, PX417342, PX417343, PX417344, PX417345, PX417346, PX417347, PX417348, PX417349, PX417350, PX417351, PX417352, PX417353, PX417354, PX417355), ranging in length from 196 to 204 bp, were obtained from parasite samples and analyzed using NCBI Blast. The alignment lengths ranged from 645 to 872 bp. Analysis revealed that the sequences were 99.46–99.93% similar to those of *Ectoplana limuli* (GenBank D85088). Detailed Blast results are shown in Table 1.

### 3.4. Phylogenetic Tree

**Figure 3:**
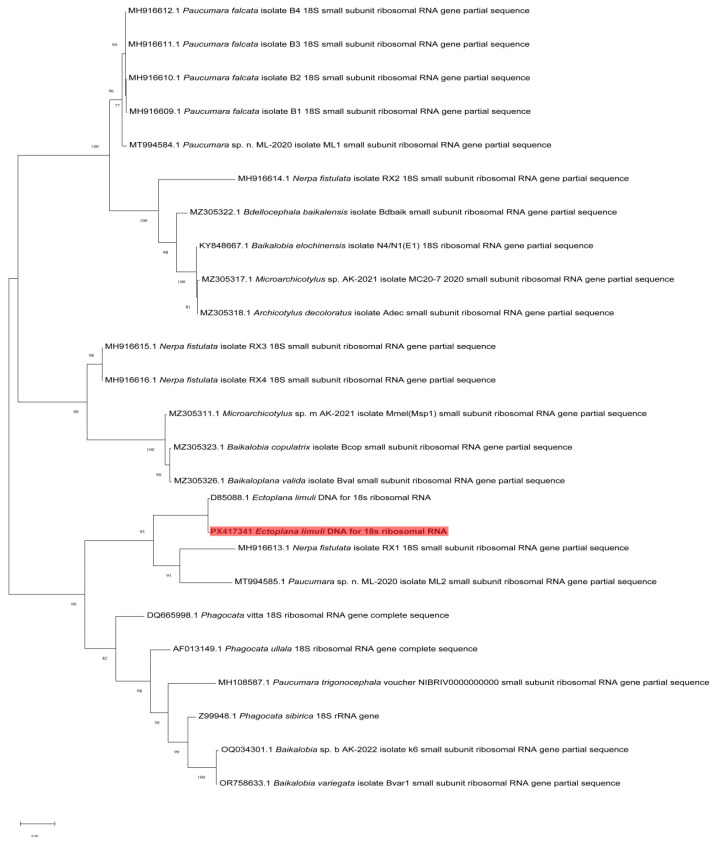
Phylogenetic tree based on the partial sequence of the 18S rDNA gene fragment, with branch support values indicating the confidence level of the nodes. Fifteen 18S rDNA gene fragment sequences were obtained, ranging in length from 196 to 204 bp; the alignment lengths ranged from 645 to 872 bp.

This study constructed a phylogenetic tree based on the 18S rRNA gene fragment, with branch support values (100, 98, 95, etc.) indicating the confidence level of the nodes (Figure 3). The phylogenetic positions of the major groups exhibited high confidence levels (Bootstrap support >90), and the phylogenetic relationships among the groups were as follows. The phylogenetic analysis strongly supported that *Ectoplana limuli* forms a well-supported clade with *Paucumara*, indicating they are sister groups. It forms a larger clade (95% support rate) with *Nerpa fistulata RX1* and *Paucumara* sp. *ML2*. *Phagocata vitta* (DQ665998.1) and *Phagocata ullala* (AF013149.1) constitute an independent branch, which is not closely related to *Ectoplana limuli*. This suggests that *Ectoplana limuli* diverged early from these turbellarian species. Species of the genus *Baikalobia* (e.g., *Baikalobia variegata*, *Baikalobia copulatix*) form an independent branch with high support (100%). The phylogenetic analysis in this study indicated that *Ectoplana limuli* belongs to a relatively independent evolutionary lineage, with a close relationship to *Nerpa fistulata* but a more distant relationship to the clades of *Paucumara* and *Baikalobia*.

## 4. Discussion

### 4.1. Morphological Characteristics of Ectoplana limuli

The organisms of the genus *Ectoplana limuli* typically exhibit a flattened body shape and possess two eyespots. The posterior end is tapered, and the mouth is ventrally located. Their bodies lack a coelom, with the distal end connecting to a typically blind-ended gut [29]. The morphological features of the parasitic species *Ectoplana limuli* investigated in the present study demonstrate a high degree of conformity with the typical characteristics of the genus *Planaria*. These shared characteristics not only support its taxonomic classification but also provide a morphological basis for investigating its adaptive evolution to a parasitic lifestyle. The outer tissue of *Ectoplana limuli* exhibits a deeply stained (purple) boundary, likely representing the epidermis or cuticle of the parasite. The intact muscle layers at the edges may appear as light purple or pink fibrous tissue under HE staining, with a certain thickness, possibly serving a protective function [30]. Observations reveal that *Ectoplana limuli* possesses only a mouth and lacks an independent anus, which aligns with the morphological characteristics of turbellarians. The digestive system has a mouth but no anus, and the complexity of its digestive tract varies. The most primitive forms lack a digestive tract, with the mouth leading to a mass of phagocytic cells (or nutritive/digestive cells) derived from the endoderm, forming a syncytium with digestive functions [31]. Sectional observations reveal some complex branched lacunar structures internally, which may represent the digestive tract or blind sacs of *Ectoplana limuli*. The digestive tract of turbellarians is sac-like or blind-tubular; in some cases, the central intestinal tract extends numerous lateral branches [31]. Certain tubular or reticular structures in the sections may represent the nervous system of *Ectoplana limuli*. The nervous system of planarians varies, with more primitive forms resembling those of cnidarians. In more advanced planarians, there is a tendency toward a reduction in the number of nerve cords, with two ventral nerve cords being the most developed, forming a primitive central nervous system (ladder-like nervous system) with the brain [32]. The overall morphology of *Ectoplana limuli* is elliptical. These morphological features not only support the classification of *Ectoplana limuli* as a distinct species but also provide important clues for understanding its ecological adaptations and evolutionary history.

### 4.2. Phylogenetic Analysis of Ectoplana limuli

The confirmation of its presence in the Liuheng sea area in Zhejiang and the Zhanjiang *T. tridentatus* Research Base suggests that this parasite may be distributed across a broader geographical range or possess a stronger environmental adaptability. Considering the migratory behavior of *T. tridentatus* [11], this parasite may have spread to multiple regions through the movement of its host, a hypothesis that warrants further validation in future studies.

In molecular identification studies of parasites, 18S rDNA is one of the commonly used molecular markers [5], known for its high reliability in identifying interspecific differences and confirming species identity [6]. When the similarity between the target sequence and the sequence of the species identified in the database is between 99.5% and 100%, the target species is considered to be the same as the matching species in the database [33]. The minor sequence differences may stem from genetic variation within the population, evolutionary divergence among parasite populations in different geographical regions [34], or microevolutionary changes induced by environmental factors such as temperature, salinity, and host health conditions [35]. Since there is currently no COI gene sequence for this parasite in the NCBI database, this analysis was not conducted in this study, and we will conduct further relevant research utilizing COI genes or whole-genome data to conduct a more in-depth analysis of the genetic diversity of this parasite in the future, thereby determining whether regional adaptation or cryptic species exist [36,37].

These findings provide new evidence for clarifying the phylogenetic position of *Ectoplana limuli*. The branch containing *Ectoplana limuli* is relatively close to that of the genus *Paucumara* (*Paucumara* sp. and *Paucumara falcata*) in the phylogenetic tree, but they do not form a monophyletic clade, suggesting a certain evolutionary relationship between them while indicating they may not belong to the same evolutionary branch. *Ectoplana limuli* occupies a relatively independent phylogenetic position, implying it may have undergone unique ecological adaptations during its evolution. Its closest relative, *Nerpa fistulata*, likely reflects a species that co-adapted to similar ecological environments, while its branch relationships with *Paucumara* and *Baikalobia* suggest that *Ectoplana limuli* may have shared a common ancestor with these groups during earlier evolutionary stages. However, *Ectoplana limuli* underwent subsequent divergence. The independent phylogenetic position of *Ectoplana limuli*, as a planarian parasitic on *T. tridentatus*, may be related to evolutionary adaptations to its parasitic lifestyle. The phylogenetic tree shows that this species does not form a monophyletic clade with free-living flatworm groups, indicating that its parasitic lifestyle may have evolved independently rather than being directly derived from free-living species.

The classic species *Bdelloura candida* of the suborder Maricola is an ectosymbiont of the American horseshoe crab (*Limulus polyphemus*) [38,39]. Its populations stably attach to the gill lamellae, appendages, and other parts of the host for a long time, and their genetic structure is highly associated with that of the host, reflecting the close symbiotic relationship resulting from co-evolution. The parasitic site selection of *Ectoplana limuli* is highly similar to the distribution pattern of *Bdelloura candida* on the body surface of American horseshoe crabs; both tend to select areas on the host’s body surface with stable water flow, less predation disturbance, and easy access to nutrients [40]. The phylogenetic tree constructed according to 18S rDNA shows that *Ectoplana limuli* is closest to *Paucumara* in the Maricola group. Although species of the genus *Paucumara* (e.g., *Paucumara falcata*) have no direct records of parasitic hosts, they are collected from intertidal benthic habitats that highly overlap with those of horseshoe crabs [41]. In the future, based on the molecular data provided in this study, we can compare the genetic differences between *Ectoplana limuli* and other Maricola species, revealing the evolutionary dynamics of parasitic relationships within this group by analyzing the host associations of different Maricola species.

Additionally, its distinct branching from the genus *Phagocata* (e.g., *Phagocata vitta* and *Phagocata ullala*) further supports the idea that its parasitic nature may have led to significant evolutionary divergence. *Ectoplana limuli* primarily inhabits marine environments [41], while *Nerpa fistulata*, *Paucumara*, and *Baikalobia* species are distributed in freshwater or low-salinity coastal environments [40,42]. This suggests that *Ectoplana limuli* may have undergone different ecological adaptation processes, resulting in a certain disconnect between its phylogenetic relationships and its morphological and niche adaptations. This phenomenon is relatively common among flatworms. For example, the protease systems of some flatworms have undergone significant changes during their adaptation to parasitic lifestyles, but these changes do not entirely preclude the possibility of their re-adaptation to free-living conditions [43]. The phylogenetic tree in this study provides some support for this hypothesis, suggesting that *Ectoplana limuli* may have originated from free-living ancestral groups and subsequently evolved different ecological adaptation strategies. Based on phylogenetic analysis, this study found that *Ectoplana limuli* shares a closer relationship with *Nerpa fistulata* and a more distant relationship with the genera *Phagocata* and *Baikalobia*. These findings provide new molecular evidence for the classification and evolution of *Ectoplana limuli* and further support the idea that it may have undergone unique ecological adaptation processes. The discovery of this planarian parasitizing *T. tridentatus* in China after more than a century raises questions about whether this is linked to the evolution of *T. tridentatus*. Future research could integrate multi-gene data and ecological niche modeling to gain a more comprehensive understanding of the role and ecological functions of *Ectoplana limuli* in the evolutionary process of *T. tridentatus*.

### 4.3. Prevalence of Ectoplana limuli in T. tridentatus

The sediment used in the breeding pond was collected from the muddy tidal flats of the natural habitat of *T. tridentatus* located near the base, and the water used for breeding was filtered and disinfected natural seawater. It is speculated that the transmission of the parasites may be related to certain specific factors in the local sediment or water quality, leading to the parasitic phenomenon in most of *T. tridentatus*. *Ectoplana limuli* was first discovered in 1916 [44]. While Kawakatsu and Sekiguchi’s study provided foundational insights into the histological structure of *Ectoplana limuli*, their work was limited in scope, focusing exclusively on tissue organization without addressing broader biological or epidemiological significance. Key gaps include (1) the absence of morphological comparisons or detailed taxonomic characterization, (2) no quantitative data on prevalence or host–parasite dynamics. Our study significantly expands upon these findings by integrating multidimensional analyses. First, we provided morphological data. Second, we quantified infection prevalence, offering ecological and epidemiological context for *Ectoplana limuli*’s impact on host populations. Most importantly, we employed molecular techniques (e.g., DNA barcoding or phylogenetic analysis) to resolve taxonomic uncertainties and enhance reproducibility for future research. By bridging these gaps, our work not only refines the understanding of *Ectoplana limuli* but also establishes a standardized framework for studying cryptic parasitic flatworms. However, more than a hundred years later, the species has been found again in different countries, which is a phenomenon worth pondering: is it because it had not been discovered before, or has the species only recently appeared in the Chinese region?

The turbellarian *Temnocephala* has been reported to heavily parasitize the gills and gill chambers of *Cherax quadricarinatus*, causing asphyxiation or bacterial and viral secondary infections in the gills, indirectly leading to mortality, which is its most significant pathogenic manifestation [45]. According to descriptions from the workers at the Zhanjiang *T. tridentatus* Research Base and fishermen from the Liuheng sea area in Zhoushan, Zhejiang, during the sampling process of this study, *Ectoplana limuli* primarily appears between June and September. The spawning season of *T. tridentatus* primarily occurs from April to September, with peak spawning activity observed from June to July [46]. During this period, suitable water temperatures (18–26 °C) and abundant nutrients facilitate rapid molting and growth of juvenile horseshoe crabs, potentially accelerating molting rates of first-instar juveniles and possibly affecting the molting frequency of juveniles. Given that June to August typically represents the warmest period, global warming may lead to an increase in parasite prevalence, as elevated temperatures can enhance parasite reproduction and host susceptibility [47]. This observation aligns with broader ecological studies demonstrating that climate change alters host–parasite dynamics, where warmer waters promote pathogen transmission and disrupt natural barriers that previously limited parasitic infections [48]. These environmental changes may render *T. tridentatus* more vulnerable to parasitic infestation during critical life stages such as molting and reproduction. Consequently, further investigation into temperature-dependent parasite–host interactions is warranted. This seasonal occurrence raises questions: how does this species die off during this period, and why does it reappear afterward? This issue warrants further exploration, especially in the context of parasite prevention and control.

### 4.4. Potential Impact of Parasitic Infections on Juvenile T. tridentatus Mortality

During the aquaculture period, it was found that juvenile *T. tridentatus* has a high mortality rate, especially for first-instar to second-instar individuals, reaching 53.75%. However, current research focuses on field resource surveys [49,50]. There is still a lack of in-depth research on the high mortality rate of juvenile *T. tridentatus* during the breeding period. Our team speculates that the high mortality rate is related to disease and nutrition. In this study, parasites were found on the body surface of spawning *T. tridentatus* in artificial breeding ponds, suggesting that parasitic infections may contribute to the elevated mortality of first-instar and second-instar individuals. This phenomenon aligns with findings in other crustaceans, such as shrimp and crabs, where parasitic infections impair host mobility, reduce feeding efficiency, and increase disease susceptibility. Notably, rising water temperatures can accelerate parasite life cycles while compromising host immune defenses [51]. A comparison of thermal performance curves (TPCs) for the parasitic dinoflagellate *Haematodinium* spp. in two crab species demonstrated that the parasite exhibits greater thermal adaptability than its hosts under elevated temperatures [52]. Similarly, in shrimp aquaculture, the ciliate *Hyalophysa lynni* lives in the gills of shrimp, triggering the host’s immune response and causing blackened nodules in the gill tissue, leading to the disease symptom commonly known as shrimp black gills [53]. Therefore, global warming may further exacerbate the parasitic infection pressure of *T. tridentatus*, forming a vicious cycle of “high temperature, high prevalence, high mortality rate”. In summary, this study is the first to explore the potential mechanism of the decline of *T. tridentatus* resources in China from the perspective of parasitic diseases. Future research should integrate long-term monitoring and experimental infection models to quantify parasite-induced mortality in juvenile *T. tridentatus* and assess how climate change (e.g., ocean warming) modulates host–parasite interactions. These findings not only advance conservation strategies for *T. tridentatus* but also offer novel insights into mitigating population declines in other endangered marine invertebrates.

## 5. Conclusions

The decline in horseshoe crab resources in China is affected by many factors. This is the first study of parasitic disease of horseshoe crab in China. This study describes the morphology and conducts molecular biological analyses of the parasites found on *T. tridentatus*, confirming that the parasites from the Liuheng marine area in Zhejiang and at the of Zhanjiang *T. tridentatus* Research Base of the South China Sea Fisheries Research Institute are the same species, *Ectoplana limuli*. *Ectoplana limuli* is reported for the first time in China and represents a new species of turbellarian in the country.

## Figures and Tables

**Table 1:** Fifteen sequences of detailed Blast results.

Sequence ID	Number of bp	Percentage
PX417341	183/184	99%
PX417342	172/173	99%
PX417343	178/178	100%
PX417344	183/184	99%
PX417345	185/186	99%
PX417346	188/194	97%
PX417347	183/184	99%
PX417348	183/184	99%
PX417349	174/175	99%
PX417350	183/184	99%
PX417351	183/184	99%
PX417352	183/184	99%
PX417353	183/184	99%
PX417354	178/178	100%
PX417355	183/184	99%

## Data Availability

Data will be made available on request.
